# Breakfast consumption, saturated fat intake, and body mass index among medical and non-medical students: a cross-sectional analysis

**DOI:** 10.1038/s41598-024-63440-x

**Published:** 2024-06-01

**Authors:** Jacksaint Saintila, Sandra P. Carranza-Cubas, Omar F. A. Santamaria-Acosta, Antonio Serpa-Barrientos, Cristian Ramos-Vera, Elmer López-López, Luis Alberto Geraldo-Campos, Yaquelin E. Calizaya-Milla

**Affiliations:** 1 Research Group for Nutrition and Healthy Behaviors, School of Medicine, Universidad Señor de Sipán, Carretera a Pimentel Km 5, Chiclayo, Lambayeque, 14001 Peru; 2 Departamento de Psicología, Universidad Nacional Mayor de San Marcos, Lima, Peru; 3 Área de Investigación, Universidad César Vallejo, Lima, Peru; 4 Universidad Tecnológica del Perú, Ica, Peru; 5 Research Group for Nutrition and Lifestyle, School of Human Nutrition, Universidad Peruana Unión, Lima 15, Peru

**Keywords:** Eating habits, Medical students, Non-medical students, Saturated fat intake, Breakfast, University students, Nutrition, Public health

## Abstract

Changes in dietary patterns and body weight have become a focus of research in undergraduate students. This study compared breakfast consumption, intake of foods high in saturated fat, and BMI between medical and non-medical students. A comparative cross-sectional study was conducted in 4,561 Peruvian university students, of whom 1,464 (32.1%) were from the medical field and 3,097 (67.9%) from the non-medical field. We compared the frequency of breakfast consumption (categorized as regular: 6 to 7 days/week; occasional: 3 to 5 days/week; and rarely or never: 0 to 2 days/week) and the frequency of consumption of foods high in saturated fat. We created simple and multiple linear and Poisson regression models with robust variance to evaluate the association of the mentioned variables with academic fields. Non-medical students (Adjusted Prevalence Ratio [PR] = 0.92, 95% CI 0.86–0.99;* p* = 0.008) were less likely to eat breakfast regularly compared to medical students. Likewise, consumption of foods high in saturated fats was higher in non-medical students (B = 1.47, 95% CI 0.91–2.04; *p* < 0.001) compared to medical students. Similarly, the mean BMI of these students was significantly higher than that of medical students (B = 0.33, 95% CI 0.12–0.53; *p* = 0.002). Although medical students reported relatively healthy eating habits and a lower BMI, there is a widespread need to promote improved diet and lifestyle among the entire university population to reduce the risks of communicable diseases and improve quality of life.

## Introduction

The transition to university life can be exciting, however, it can be challenging for many students, who are forced to continuously adapt to fluctuations in academic load, which includes the volume and intensity of academic work, such as courses, homework, exams, and other academic responsibilities^1,2^. More specifically, this adaptation often has a negative impact, leading to significant changes in lifestyle, including the adoption of unhealthy eating patterns, such as the tendency to skip breakfast and the consumption of foods rich in saturated fats, which are associated with a high risk of overweight and obesity^3^.

Breakfast, which is generally consumed within the first two hours after awakening, is considered the most important meal of the day, due to its role in providing energy and essential nutrients to start daily activities^3,4^. Several studies indicate that breakfast has a positive effect on nutritional balance and cognitive functioning, due to better attention and concentration, which influences academic performance^5–8^. However, despite the advantages it offers, skipping breakfast is a common habit among university students^4,9–11^. For example, a study reported that 49.8% skipped breakfast^9^. Another study that evaluated 92 medical students found that, among the three meals of the day, breakfast was the least consumed by 54.4%^11^. Furthermore, another study noted that one in three university students skips breakfast^4^. However, some studies reported significant differences between medical and non-medical students, showing a higher breakfast consumption among medical students^12,13^. It is important to emphasize that the omission of this food increases the risk of obesity by 43%^3^.

On the other hand, university students tend to consume foods rich in saturated fats^14^. In the university population of developed countries, a systematic review indicates that the prevalence of fat, fast food, and ultra-processed food consumption is 45.61%^15^. Similarly, a study conducted among students in the United Arab Emirates showed that 48% of students had a high intake of total fats, 90% consumed saturated fats, and 64% trans fats^16^. In the context of Latin America, a study conducted in 10 countries in the region reported a high consumption of ultra-processed foods among university students^17^. High consumption of these foods has a negative impact on health, leading to an increased risk of non-communicable diseases such as obesity, atherosclerosis, insulin resistance, type 2 diabetes, cerebrovascular disease, myocardial infarction, sleep disorders, and different types of cancer^14,15^. Additionally, the consumption of healthy foods such as fruits and vegetables are often low^9,15^. For example, among non-medical students, only 10.17% consume fruits and vegetables daily^18^. Concurrently, 25.42% consume processed meats at least once a week, while 37.29% consume fast food, foods that are high in saturated fats^18^.

Regarding the difference between medical and non-medical university students, studies indicate that the consumption of red meat and fast foods, such as french fries, pizza, and hamburgers, was more frequent among non-medical students, as well as other ultra-processed products high in saturated fats, such as cookies, cakes, chocolates, chips, or sticks^19^. These results could be explained considering that medical students tend to have a deeper and more detailed knowledge about healthy lifestyle habits compared to students in other disciplines^20^. This is because their academic training includes a significant focus on health, nutrition, and wellness issues, which likely influences their healthy food choices and practices.

Overweight and obesity pose a public health challenge for young people in general and university students in particular. In Peru, based on current data from the National Institute of Statistics (INEI), 37.5% of individuals aged 15 and above are overweight, while 25.6% are obese^21^. Obesity rates are higher in urban areas and among women, with 39.0% and 29.8% respectively^21^. Which places Peru among the countries with the highest rates of non-communicable diseases in the Latin American region. Based on the BMI categorization established by the World Health Organization (WHO), a study found that 20% of students were overweight.

The proportion of excess body weight was higher among students from non-medical universities compared to those from medical fields^19^. Similarly, a study showed that a lower percentage of medical students (28.7%) had a BMI ≥ 25 compared to students in other academic disciplines (37.9%)^22^. A high BMI among university students highlights the necessity of a comprehensive approach to addressing physical and psychological health. This can be achieved by promoting a multidisciplinary approach that includes health education, access to wellness resources, and a university environment that supports healthy lifestyles.

Poor habits and increased body weight among university students remain one of the most significant concerns in the field of public health. However, there is a scarcity of studies comparing dietary habits such as regular breakfast consumption and the intake of foods rich in saturated fat and BMI in this population group. Although several studies have examined eating behaviors and nutritional status of university students; relatively few studies have focused on studying eating habits among medical and non-medical students^23–26^. Understanding these nutritional aspects between both groups is important to support the implementation of interventions aimed at reducing poor dietary practices and reducing the risk of communicable disease^16^. Therefore, the present study aims to analyze and compare breakfast consumption, intake of foods rich in saturated fat and BMI between medical and non-medical students.

## Materials and methods

### Study design and participants

This cross-sectional comparative study was designed to evaluate and compare two groups of university students, differentiated by their field of study: (1) medical science students, which include those enrolled in Nursing, Nutrition and Dietetics, and Medicine, and (2) students from non-health related fields, such as Theology, International Commerce, Business Administration, Engineering and Architecture, and Human Sciences and Education, among others. The research was carried out on the three campuses of the Universidad Peruana Unión, an educational institution of the Seventh-day Adventist Church located in three cities (Lima, Juliaca, and Tarapoto, coinciding with the enrollment period of the first academic year 2022, specifically during the months of February and March.

**Figure 1:**
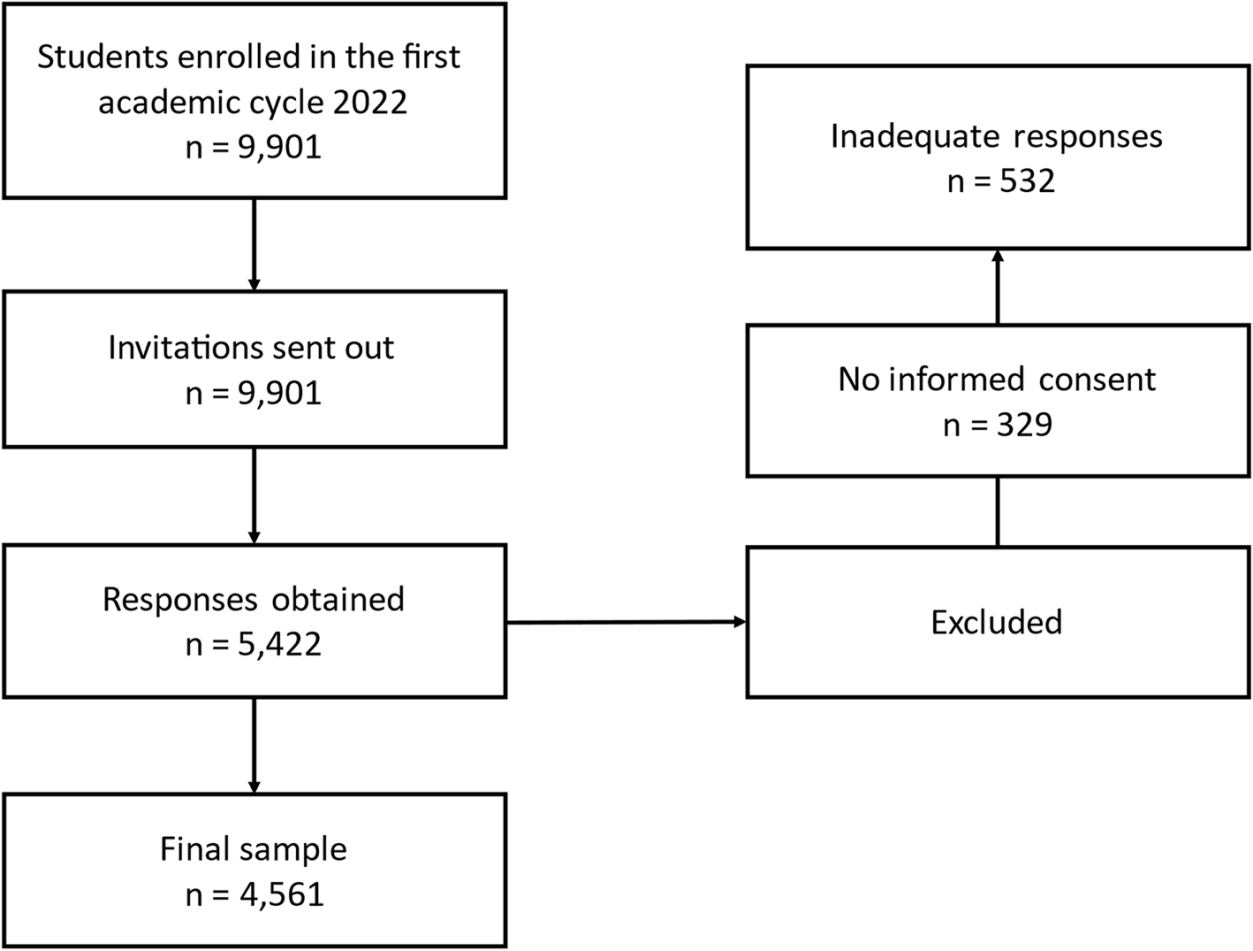
Composition of the sample.

The selection of participants was based on a convenience sample, using the university's academic portal as the main invitation method, where detailed information on the purposes and scope of the study was published. This information was available on the survey home page to ensure that all potential participants had a clear understanding of what their participation entailed. The inclusion criteria focused on adult students between 18 and 29 years old. Those outside the established age range, post-graduate students, individuals who provided inadequate responses to the survey (for example, those that are incomplete and do not follow the instructions provided in the survey), and those who did not give their informed consent to participate were excluded from the study (See Fig. 1).

### Ethical considerations

Before the start of data collection, informed consent was obtained electronically, ensuring that student participation was completely voluntary and based on a clear understanding of their participation in the study. The ethical approval of the research protocol was granted by the Research Ethics Committee of the Universidad Peruana Unión, under approval number 2022-CEUPeU-0009. The conduct of the study was rigorously aligned with international ethical standards, following the principles dictated in the Declaration of Helsinki and its subsequent amendments.

### Variables

#### Breakfast consumption

To determine the frequency with which the participants ate breakfast, the following standard question was applied: *How often do you eat breakfast per week?* This question has been used in previous research^27–29^. Participants were asked to indicate, through a numerical response of 0 to 7, the number of days per week that they usually had breakfast. Based on their responses, the frequency of breakfast consumption was classified into three different levels: those who had breakfast "rarely or never" did so between 0 and 2 days a week; the "occasional" group included those who had breakfast between 3 and 5 days a week; and "regular" was considered those who had breakfast between 6 and 7 days a week^28,29^.

#### Consumption of foods high in saturated fats

To assess the habitual intake of foods rich in saturated fats among the participants, a previously designed and validated food frequency questionnaire was used^30^. This instrument was adapted and validated for the Peruvian university population and consists of 16 items^1^. The assessment of the consumption of these foods was carried out using scales that assign points based on frequency: "never or 1 time per month or less" is assigned 0 point; "2 to 3 times per month" equals 1 point; "1 to 2 times a week" is considered with 2 points; "3 to 4 times a week" is awarded 3 points; and consumption "5 or more times a week" receives 4 points. Therefore, a lower score indicates a very low fat intake, while a higher score reflects a high fat intake. The reliability of the questionnaire was confirmed with coefficient values greater than 0.90, including an ordinal alpha of 0.94, an omega of 0.94 and an H of 0.95, demonstrating its high reliability and suitability to measure saturated fat intake in this population group^1^.

#### BMI

Data on the weight and height of the students were collected through self-reporting within the study's framework. The BMI was calculated for each participant using the provided information. BMI categorization was performed according to the World Health Organization (WHO) guidelines, which establish different weight categories. The category 'underweight' is identified with a BMI less than 18.5; "Normal weight" is defined as having a BMI ranging from 18.5 to 24.9 kg/m^2^; the range classified as 'overweight' is between 25.0 and 29.9 kg/m^2^, while 'obesity' is defined as having a BMI of 30 kg/m^2^ or higher^31^.

#### Sociodemographic information

Sociodemographic information was collected using a set of six questions that covered various essential categories for the study. These categories included aspects such as age, sex, marital status, and religion. In addition, the field of study was considered: students of health sciences (Nursing, Nutrition and Dietetics, and Medicine) and students of non-health fields (Theology, International Commerce, Business Administration, Engineering and Architecture, and Human Sciences and Education, among others).

### Statistical analysis

The first activity of the statistical analysis consisted of evaluating the validity and reliability of the instruments used to ensure the rigor with which the variable is measured in the study sample. Subsequently, a descriptive analysis was performed to describe categorical and numerical variables using absolute and relative frequencies and mean with standard deviation, respectively. Furthermore, we compared general characteristics, breakfast consumption, and intake of foods rich in saturated fats according to the study fields. To determine the existence of statistically significant differences between groups in our independent variables, we applied the Student's t-test to numerical variables and the chi-square test of independence to categorical variables. Finally, we create linear regression models and Poisson regression model (Prevalence Ratios) with robust variance to investigate the association between regular breakfast consumption, intake of foods high in saturated and BMI with academic fields, adjusting for age, sex, and parental education. A threshold of statistical significance was established with a value of *p* < 0.05 for all tests performed. Data analysis was performed using R statistical software, version 4.3.2^32^.

### Ethics approval and consent to participate

Informed consent was obtained electronically, ensuring that student participation was completely voluntary and based on a clear understanding of their involvement in the study. Ethical approval of the research protocol was granted by the Research Ethics Committee of the Universidad Peruana Unión, under approval number 2022-CEUPeU-0010. The conduct of the study was rigorously aligned with international ethical standards, following the principles dictated in the Declaration of Helsinki and its subsequent amendments.

## Results

The average age of the participants was 21.4 years. Females represented 74.7% of students in medical fields. Significantly more medical students were single (95.1%) and professed the Adventist religion (58.7%). Regular breakfast consumption is associated with the medical field (*p* = 0.024). Non-medical students were more likely to report the consumption of foods high in saturated fat (*p* < 0.001). Additionally, these students had a higher mean score (18.25) in the consumption of foods rich in saturated fats than medical students (16.39;* p* < 0.001). The BMI categories differed significantly between the groups (*p* = 0.040), indicating that 25% of students in the non-medical group had excess body weight with a slightly higher mean score (23.8) compared to the medical group (23.5;* p* = 0.004) (Table 1).

**Table 1:** Sociodemographic characteristics, breakfast consumption, intake of foods high in saturated fats, and BMI according to academic fields.

Characteristics	All N = 4,561		Medical, n = 1,464 (32.1%)		Non-Medical n = 3,097 (67.9%)		*p*^a^
	n	%	n	%	n	%	
**Age (M ± SD)**	21.5	3.4	21.3	3.1	21.7	3.5	0.153
**Age (years)**							< 0.001
18–19	879	19.3	399	27.3	480	15.5	
20–24	3,004	65.9	873	59.6	2,131	68.8	
25–29	678	14.8	192	13.1	486	15.7	
**Sex**							< 0.001
Female	2,481	54.4	1,094	74.7	1,387	44.8	
Male	2,080	45.6	370	25.3	1,710	55.2	
**Education of parents**							0.059
None/Primary	1,636	35.9	530	36.2	1,106	35.7	
High school	975	21.4	275	18.8	700	22.6	
Technical	811	17.8	250	17.1	561	18.1	
Bachelor's Degree	704	15.4	255	17.4	449	14.5	
Postgraduate	435	9.5	154	10.5	281	9.1	
**Religious affiliation**							0.010
Seventh-day Adventist	2,637	57.8	859	58.7	1,778	57.4	
Non-Adventist	1,924	42.2	605	41.3	1,319	42.6	
**Marital status**							< 0.001
Married	276	6.1	72	4.9	204	6.6	
Singles	4,285	93.9	1,392	95.1	2,893	93.4	
**Breakfast consumption**							0.024
Regular (6 to 7 days/week)	2,536	55.6	848	57.9	1,688	54.5	
Occasional (3 to 5 days/week)	1,282	28.1	384	26.2	898	29	
Rarely or never (0 to 2 days/week)	743	16.3	232	15.9	511	16.5	
**Consumption of foods high in saturated fats**							< 0.001
Low	302	6.2	107	7.3	195	6.3	
Medium	2,424	53.2	851	58.1	1,573	50.8	
High	1,835	40.4	506	34.6	1,329	42.9	
**Saturated fat consumption score (M ± SD)**	17.4	2.8	16.4	2.5	18.3	3.1	< 0.001
**BMI (Categories)**							0.040
Underweight	119	2.6	44	3.0	75	2.4	
Normal	3,139	68.7	1,033	70.6	2,106	68.0	
Obesity	194	4.3	53	3.7	141	4.5	
Overweight	1,109	24.4	334	22.8	775	25.0	
**BMI score (M ± SD)**	23.6	3.2	23.5	3.1	23.8	3.2	0.004

M: Mean, SD: Standard Deviation; BMI: Body mass index.

^a^Student's t-test and chi-square test of independence.

Table 2 showed significant differences in the categories of consumption of hamburgers, ground beef, meat burritos, or tacos (*p* < 0.001); Beef or pork, such as steaks, roasts, ribs, or sandwiches (*p* < 0.001); fried chicken (*p* < 0.001); Hot dogs, or Polish or Italian sausage (*p* < 0.001); Cold cuts, lunch meats, ham (not low fat) (*p* < 0.001); Bacon or breakfast sausage (p < 0.001); Salad dressings (not low fat) (*p* < 0.001); Margarine, butter or mayo on bread or potatoes (*p* = 0.001); Pizza (*p* < 0.001); Cheese, cheese spread (not low fat) (*p* = 0.005); Whole milk (*p* = 0.034); French fries, fried potatoes (*p* < 0.001); Doughnuts, pastries, cake, cookies (not low-fat) (*p* < 0.001); and Ice cream (not sherbet or non-fat) (*p* < 0.001). In general, analyzing the highest frequencies of weekly consumption (5 or more times/week, 3–4 times/week, and 1–2 times/week), non-medical students have slightly higher percentages of frequent consumption in all food categories mentioned compared to medical students.

**Table 2:** Frequency of consumption of foods rich in saturated fats in medical and non-medical students.

Food items	Medical, n = 1,464 (32.1%)		Non-Medical n = 3,097 (67.9%)		*p*^*a*^
	n	%	n	%	
**Hamburgers, ground beef, meat burritos, tacos**					< 0.001
1/month or less	403	27.5	669	21.6	
2–3 times/month	813	55.5	1,759	56.8	
1–2 times/week	170	11.6	453	14.6	
3–4 times/week	55	3.8	142	4.6	
5 or more times/week	23	1.6	74	2.4	
**Beef or pork, such as steaks, roasts, ribs, or in sandwiches**					< 0.001
1/month or less	423	28.9	684	22.1	
2–3 times/month	728	49.7	1,601	51.7	
1–2 times/week	195	13.3	505	16.3	
3–4 times/week	83	5.7	208	6.7	
5 or more times/week	35	2.4	99	3.2	
**Fried chicken**					< 0.001
1/month or less	126	8.6	206	6.7	
2–3 times/month	895	61.2	1,781	57.5	
1–2 times/week	300	20.5	722	23.3	
3–4 times/week	103	7.1	273	8.8	
5 or more times/week	40	2.7	115	3.7	
**Hot dogs, or Polish or Italian sausage**					< 0.001
1/month or less	637	43.5	1,053	34	
2–3 times/month	634	43.3	1,474	47.6	
1–2 times/week	127	8.7	369	11.9	
3–4 times/week	47	3.2	136	4.4	
5 or more times/week	19	1.3	65	2.1	
**Cold cuts, lunch meats, ham (not low-fat)**					< 0.001
1/month or less	618	42.2	1,050	33.9	
2–3 times/month	640	43.7	1,471	47.5	
1–2 times/week	139	9.5	375	12.1	
3–4 times/week	48	3.3	136	4.4	
5 or more times/week	19	1.3	65	2.1	
**Bacon or breakfast sausage**					< 0.001
1/month or less	862	58.9	1,552	50.1	
2–3 times/month	436	29.8	139	34.5	
1–2 times/week	104	7.1	310	10	
3–4 times/week	41	2.8	102	3.3	
5 or more times/week	21	1.4	65	2.1	
**Salad dressings (not low-fat)**					< 0.001
1/month or less	430	29.4	719	23.2	
2–3 times/month	754	51.5	1,675	54.1	
1–2 times/week	165	11.3	461	14.9	
3–4 times/week	84	5.7	161	5.2	
5 or more times/week	31	2.1	81	2.6	
**Margarine, butter or mayo on bread or potatoes**					0.001
1/month or less	329	22.4	613	19.8	
2–3 times/month	858	58.5	1,802	58.2	
1–2 times/week	187	12.8	452	14.6	
3–4 times/week	64	4.4	161	5.2	
5 or more times/week	26	1.8	71	2.3	
**Margarine, butter, or oil for frying eggs (not Egg Beaters or just egg whites)**					0.144
1/month or less	340	23.2	677	21.8	
2–3 times/month	805	55.0	1,675	54.1	
1–2 times/week	205	14.0	592	15.9	
3–4 times/week	85	5.9	180	5.8	
5 or more times/week	29	2.0	73	2.4	
**Pizza**					< 0.001
1/month or less	394	26.9	737	23.8	
2–3 times/month	912	62.3	1,914	61.8	
1–2 times/week	107	7.3	294	9.5	
3–4 times/week	32	2.2	99	3.2	
5 or more times/week	19	1.3	53	1.7	
**Cheese, cheese spread (not low-fat)**					0.005
1/month or less	278	19.0	486	15.7	
2–3 times/month	799	54.6	1,743	56.3	
1–2 times/week	242	16.5	545	17.6	
3–4 times/week	105	7.2	242	7.8	
5 or more times/week	40	2.7	81	2.6	
**Whole milk**					0.034
1/month or less	313	21.4	592	19.1	
2–3 times/month	772	52.7	1,660	53.6	
1–2 times/week	252	17.2	526	17	
3–4 times/week	89	6.1	229	7.4	
5 or more times/week	38	2.6	90	2.9	
**French fries, fried potatoes**					< 0.001
1/month or less	108	7.4	195	6.3	
2–3 times/month	976	66.7	1,933	62.4	
1–2 times/week	283	19.3	656	21.2	
3–4 times/week	74	5.0	232	7.5	
5 or more times/week	23	1.6	81	2.6	
**Corn chips, potato chips, popcorn, crackers**					0.049
1/month or less	208	14.2	387	12.5	
2–3 times/month	950	64.9	1,979	63.9	
1–2 times/week	212	14.5	517	16.7	
3–4 times/week	69	4.7	155	5	
5 or more times/week	25	1.7	59	1.9	
**Doughnuts, pastries, cake, cookies (not low-fat)**					< 0.001
1/month or less	228	15.6	461	14.9	
2–3 times/month	981	67.0	1,964	63.4	
1–2 times/week	173	11.8	458	14.8	
3–4 times/week	60	4.1	146	4.7	
5 or more times/week	22	1.5	68	2.2	
**Ice cream (not sherbet or non-fat)**					< 0.001
1/month or less	211	14.4	400	12.9	
2–3 times/month	1,025	70.0	2,112	68.2	
1–2 times/week	165	11.3	396	12.8	
3–4 times/week	45	3.1	121	3.9	
5 or more times/week	18	1.2	68	2.2	

^a^chi-square test of independence.

Non-medical students (Adjusted PR = 0.92, 95% CI: 0.86 – 0.99;* p* = 0.008) were significantly less likely to have breakfast regularly compared to those in the medical field. Similarly, in relation to fat intake, non-medical students (B = 1.47, 95% CI: 0.91 – 2.04; *p* < 0.001) are more likely to have a high consumption of foods rich in saturated fats compared to those of medical specialties. Similarly, medical students have a significantly lower mean BMI than non-medical students (B = -0.33, 95% CI: -0.53—-0.12; p = 0.002) (Table 3).

**Table 3:** Simple and multiple regression models of regular breakfast consumption and intake of foods high in saturated fat.

	Simple regression			Multiple regression^a^		
	Regular breakfast consumption					
**Academic Field**	PR	95% CI	*p*	PR	95% CI	*p*
Medical	Ref			Ref		
Non-medical	0.93	0.87–0.99	**0.010**	0.92	0.86–0.99	**0.008**
	**Score of intake of foods high in saturated fat**					
**Academic Field**	B	95% CI	*p*	B	95% CI	*p*
Medical	Ref			Ref		
Non-medical	1.62	1.05–2.18	** < 0.001**	1.47	0.91–2.04	** < 0.001**
	**BMI**					
**Academic Field**	B	95% CI	p	B	95% CI	p
Medical	Ref			Ref		
Non-medical	0.35	0.15–0.56	**0.001**	0.33	0.12–0.53	**0.002**

Linear regression was used for the intake of foods high in saturated fat score, and Poisson regression with robust variance was used for the regular breakfast consumption variable.

PR: Prevalence Ratios; B: Regression coefficient; BMI: body mass index.

^a^Adjusted for age, sex, and parental education.

## Discussion

This comparative cross-sectional analysis not only sheds light on the dietary preferences of future health professionals and those in other academic disciplines, but also invites reflection on the implications of such choices. Although breakfast habits can reveal trends toward a more or less healthy lifestyle, the intake of foods rich in saturated fats and BMI provide a picture of risks and behaviors associated with non-communicable diseases, which are of particular interest in the field of public health^33^. The results of the study indicate that: (a) compared to medical students, those studying other disciplines have a lower tendency to consume breakfast regularly; (b) it is observed that students from disciplines other than health sciences tend to have a higher frequency of consumption of foods with high levels of saturated fats; and (c) similarly, it was found that the average BMI is significantly higher in students who do not study medical sciences compared to their peers in the medical field.

### Regularity of breakfast consumption

Regular breakfast consumption contributes significantly to daily intake of essential nutrients, such as vitamins and minerals, including calcium, iron, and B vitamins^34^. Scientific evidence and current recommendations for daily breakfast intake from national and international organizations, such as the Peruvian Ministry of Health and the Academy of Nutrition and Dietetics, agree that breakfast is an effective way to start the day, providing the energy and nutrients essential for optimal performance in both children and adults^35,36^. Additionally, they highlight the importance of including a variety of foods in breakfast, such as whole grains, fruits, lean proteins, and low-fat or non-fat dairy products^35,36^. Moreover, regular breakfast consumption has been associated with improved cognitive performance, due to enhanced attention, concentration, and memory, especially among school and university populations^5,6^. However, despite the benefits associated with regular breakfast consumption, the tendency to skip this meal is common among the university population.

In the current study, it was found that non-medical students exhibit a lower tendency to eat breakfast regularly. These findings are consistent with the results of a study in which 18,231 students in Mongolia were evaluated, revealing that medical students reported higher breakfast consumption than non-medical students^12^. Following that same line of thought, recently, a cross-sectional survey of 839 university students in Pakistan found that a higher proportion of non-medical students reported skipping breakfast (30.23% vs. 26.96%) compared to their health sciences counterparts^37^. It is worth mentioning that these differences can be framed in the perception of the importance of nutrition and wellness among students in health sciences and other academic fields. It is possible that medical students, due to their training in health sciences, may be more aware of the importance of maintaining healthy eating habits, including regular consumption of breakfast^13,37^. For example, in a study in which participants were asked if breakfast was an essential component of their daily diet, 57.4% of medical students responded affirmatively; however, 65% of non-medical students did not, demonstrating a significant difference between the two groups^13^.

On the other hand, we found that more than 44% of the students reported skipping breakfast. These findings are consistent with previous studies. For example, research among Chilean university students aged 18 to 27 years of age in various fields revealed that 47% of them did not regularly eat breakfast^3^. Similarly, a study conducted among medical and biomedical science students found that 49.8% skipped breakfast^9^.

Furthermore, another study evaluating a group of medical students at a Peruvian university highlighted that breakfast was the least consumed meal, skipped by 54.4% of the participants^11^. Additionally, a study conducted in medical students indicated that the overall rate of breakfast omission reached 71.92%^10^.

While medical students may be more inclined to regularly consume breakfast due to their increased awareness of health, these findings nonetheless highlight a concerning trend toward skipping breakfast among the university student population at large, suggesting a potential area of intervention to improve eating habits. In fact, there is a clear need to expand nutritional education and promote healthy eating habits among students from all disciplines. The implementation of strategies and programs focused on the importance of breakfast could contribute significantly to the academic performance of the student population in general^5,6^. Furthermore, the need to address breakfast skipping is highlighted, as it is associated with several negative health effects, including an increased risk of obesity and metabolic diseases^28^.

### Consumption of foods high in saturated fats

Consumption of foods rich in saturated fats, trans fats and cholesterol has been associated with an increased probability of developing cardiovascular disease, because it increases serum cholesterol levels in the blood^38^. In response to the risks associated with the intake of these foods, international organizations, such as the American Heart Association, advise that adults keep their consumption of saturated fats below 7% of total daily calories, trans fats below 1% of total daily calories and cholesterol to less than 300 mg per day^39,40^. Similarly, the 2010 Dietary Guidelines for Americans recommend limiting the intake of sources of saturated and trans fats, including products such as butter, beef fat, poultry fat, pork fat (lard), solid margarine and vegetable shortening^41^. In Peru, in June 2019, the Ministry of Health published the Manual of Advertising Warnings as part of Law No. 30021, the Law for the Promotion of Healthy Eating, which requires front-of-package labeling on processed food products that are high in saturated fats and contain trans fats^42^. These recommendations aim to promote a healthy lifestyle and decrease the consumption of foods high in saturated fats by encouraging dietary habits that reduce the risk of cardiovascular diseases.

The current findings reveal that non-medical students tend to have a higher frequency of consumption of foods rich in saturated fats. In our study we evaluated the frequency of consumption of foods rich in saturated fats such as fried chicken, hot dogs, french fries, pizza, hamburger, red meat, among others. The findings of our study are in line with a study that reported that the consumption of red meat and fast foods, such as French fries, pizza, and hamburgers, was consumed more frequently in nonmedical students, as was other ultraprocessed products, such as cookies, cakes, chocolates, chips, or sticks^19^, which are rich in saturated fats and contain trans fats^43^. Similarly, in a recent study that explored food consumption patterns among university students, when asked about their purchasing habits at fast food restaurants, it was found that a significantly higher proportion of non-medical students reported buying this type of food, with 61.19% compared to 57.51% of medical students^37^. These results could be explained considering that medical students tend to have a deeper and more detailed knowledge about healthy lifestyle habits compared to students in other disciplines^20^.

However, it is important to note that previous findings showed that students in both medical and non-medical fields showed elevated rates of fast-food consumption, which are a source of saturated and trans fats^19,37^. In our study, the overall scores for the consumption of foods high in saturated fats were 17.45, which equates to a moderately high level of consumption. On the other hand, interestingly, the results of one study reported that fast food consumption was more frequent among medical students than non-medical students^13^. This indicates that, despite the differences observed, the tendency to opt for fast food is significantly high among the university population in general. This pattern suggests the existence of common factors, such as convenience, lack of time to prepare healthy foods, or lack of knowledge about healthy eating, that could influence students' food choices, regardless of their field of study.

It is important to highlight that while knowledge about healthy eating habits is essential, it does not automatically lead to their adoption^44,45^. To promote healthy eating among university students, it is essential to address the underlying barriers, as well as to encourage a healthier food environment and to promote strategies that facilitate more nutritious and balanced food choices. In any case, the high proportion of students who resort to foods high in saturated fats highlights the urgent need to address these issues through health and nutrition education, as well as to promote healthy food alternatives in the university environment. It should be specified that, although we have not evaluated fast food per se in the current study, foods such as hamburgers, french fries, pizza, fried chicken, hot dogs, and cakes are identified as the most common foods found in fast food restaurants.

### BMI

High BMI in undergraduate students, whether medical or non-medical, presents various challenges and considerations for overall health and well-being. The scientific literature has established a clear relationship between a high BMI and an increased risk of developing non-communicable diseases, such as type 2 diabetes, cardiovascular disease, and certain types of cancer^46^. This risk, spread over time, can significantly impact not only students' quality of life but also their academic performance and future careers^47,48^. Our research consistently shows that students enrolled in non-medical schools have a significantly higher average BMI compared to those in medical fields. This observation is in line with the existing literature, which indicates a significant prevalence of overweight among the student population, especially among those enrolled in non-medical fields^19,22^. Previous research has shown that around 20% of university students are overweight, with a higher prevalence of excess weight observed among students in non-medical fields^19^. Furthermore, a study found that the proportion of students with a BMI ≥ 25 was lower in medical schools (28.7%) compared to students from other disciplines (37.9%)^22^.

Despite these trends, some studies have found no statistically significant differences in average BMI values between students in medical specialties and those in other academic areas^25,49,50^. In fact, interestingly, in certain investigations, the average BMI of medical science students was slightly higher^25,49,50^. These differences can be attributed to various contextual and methodological factors, such as variations in lifestyles, access to health and nutrition information, and the population sample studied. The results' variability emphasizes the complexity of the factors influencing BMI and the necessity for comprehensive approaches to better comprehend these patterns in diverse college populations.

### Limitations and future research

The interpretation of the results obtained in this study is subject to several limitations that must be considered. First, the research was conducted within a specific university context, involving students from a single institution. This implies that the findings do not necessarily reflect the dietary trends and behaviors of the general population of university students, thus limiting their applicability and generalizability to broader contexts. Given this scenario, future research is essential to broaden the scope of study, including more diverse and representative samples of students from various universities and geographical contexts.

Second, in the current study, weight and height were self-reported, which may introduce bias or error, such as the flat slope-syndrome, where heavier individuals tend to underestimate their weight and shorter individuals tend to overestimate their height. However, to minimize risks in our study, we implemented several strategies. These included emphasizing to participants the importance of providing accurate information, assuring them of confidentiality, and explaining how their data would contribute significantly to the research. Additionally, we offer comprehensive and standardized guidelines for measuring weight and height. This approach aimed to enhance accountability and accuracy in the responses provided.

Third, both breakfast consumption and the frequency of consumption of foods high in saturated fat were based on self-reported reports by participants. This approach is susceptible to response biases, as participants may have difficulty accurately recalling their eating habits or may tend to present a more favorable picture of their eating behavior. Therefore, it is important that future research employ more objective and accurate methods to collect data on dietary patterns, such as the use of validated food diary or tracking of the diet through mobile applications.

### Implications for public health

Although the present study has certain limitations, we believe that the results obtained are of considerable importance, particularly in terms of public health and the development of health-based education and prevention policies. The study findings highlight several public health concerns that deserve attention. On the one hand, the fact that non-medical students show less regularity in breakfast consumption suggests an opportunity for educational interventions aimed at improving the eating habits of this population. The importance of breakfast in the maintenance of a healthy metabolism and in the prevention of noncommunicable diseases is well documented, which makes it essential to encourage its regular consumption among all university students, regardless of their field of study.

On the other hand, the tendency of non-medical students to consume foods with high levels of saturated fats highlights the need for nutrition education programs that promote greater awareness of the consequences of a diet rich in this type of fat. These programs could focus on teaching about the risks associated with excessive consumption of saturated fats, including cardiovascular disease and obesity, and offer practical strategies to adopt a more balanced diet.

Finally, the finding that the average BMI is significantly higher among non-medical students highlights a critical concern regarding obesity and overweight, conditions that are risk factors for numerous noncommunicable diseases. This suggests the importance of designing and implementing student wellness programs that include physical activity and weight management education components, especially for those in non-medical disciplines. These programs could help students maintain a healthy BMI and understand the importance of an active lifestyle and proper nutrition, contributing to the prevention of lifestyle-related diseases.

## Conclusion

This cross-sectional study compared breakfast consumption and intake of foods high in saturated fats between medical and non-medical students. It was found that non-medical students exhibited a lower tendency to regularly consume breakfast compared to medical students. In addition, it is observed that non-medical students tend to have a higher frequency of consumption of foods high in saturated fats. Similarly, non-medical students had a significantly higher average BMI compared to their peers in the medical field. Despite the relatively healthy eating habits (regular breakfast consumption and lower intake of foods high in saturated fats) and a lower BMI observed among medical specialty students, there is a widespread need to promote improvements in nutrition and lifestyle across the entire university population to reduce the risks of noncommunicable diseases and improve quality of life.

## Data Availability

The datasets generated to support the findings of this study are not publicly accessible due to ethical and legal constraints imposed by the Research Ethics Committee of the Universidad Peruana Unión. However, interested parties may request access to these datasets via email to the Ethics Committee at etica@upeu.edu.pe or to the university's General Research Directorate at director.dgi@upeu.edu.pe.
